# Case report: Application of morphology in the diagnosis of siderosis in a patient with tuberculosis infection

**DOI:** 10.3389/fonc.2023.1001802

**Published:** 2023-02-02

**Authors:** Yuli Zhou, Ying Wang, Wenbing Liu, Haibin Wang, Daqiang He, Juan Jin, Qiaoyun Li, Junying Li, Qiong Chen, Senlin Ruan, Shenghai Wu, Jiyu Tang

**Affiliations:** ^1^ Department of Laboratory, Hangzhou First People’s Hospital, Zhejiang University School of Medicine, Hangzhou, China; ^2^ Department of Laboratory, Shenzhen Second People’s Hospital, Shenzhen, China; ^3^ Department of Cardiopulmonary Medicine, Zhejiang Rehabilitation Medical Center, Hangzhou, China; ^4^ Department of Radiology, Hangzhou First People’s Hospital, Zhejiang University School of Medicine, Hangzhou, China; ^5^ Hangzhou Medical College, Lin’an people’s Hospital, Hangzhou, China; ^6^ Department of Pathology, Hangzhou First People’s Hospital, Zhejiang University School of Medicine, Hangzhou, China; ^7^ Agricultural and Biological Ring Testing Center, Zhejiang University, Hangzhou, China; ^8^ Department of Nephrology, Zhejiang Provincial People’s Hospital, Hangzhou Medical College, Zhejiang Chinese Medical University, Hangzhou, China

**Keywords:** welder, siderosis, tuberculosis, morphology, case report

## Abstract

A 49-year-old male who had been working in welding for more than 30 years was admitted to the hospital for a medical checkup that revealed a lung shadow without specific symptoms such as coughing and sputum. Imaging studies showed diffuse ground-glass changes in both lungs, wall cavities with wall nodules, multiple peripheral nodules, and some nodules with calcification. The patient has been engaged in welding work for more than 30 years and exposed to iron dust. Lung tissue biopsy, routine morphological and pathological fluid basis examination of alveolar lavage fluid, can be considered as pulmonary iron particles, which can be regarded as iron dust lung. Acid-fast bacilli were detected in both fibrobronchoscopic brush extract and alveolar lavage fluid acid-fast staining. As the pathological examination revealed granulomatous inflammation showed caseation necrosis, the patient was judged to have concomitant pulmonary TB. After the diagnosis was made, the patient was no longer exposed to dust and was treated with appropriate anti- tuberculosis (TB) therapy. Lung lesions caused by welding have been reported, but the simultaneous finding of siderosis with pulmonary TB is specific to the case presented here. By describing the imaging features, combining different staining methods of alveolar lavage fluid and pathological examination of lung tissue, we showed various morphological manifestations of this case, aiming at improving the morphological diagnosis level of laboratory physicians and enabling patients to be diagnosed and treated early.

## Introduction

At first described by Zenker in 1866 (1), iron dust lung is a rare occupational disease characterized by the accumulation of iron particles within the alveolar macrophages as a result of iron oxide in welding fumes, especially in welders, generally without associated symptoms or dysfunction. When the lungs are examined at autopsy, large deposits of iron oxide are observed yet there is no fibrosis. This condition is called siderosis and is typically classed as benign pneumoconiosis, but patients can develop chronic bronchitis and interstitial fibrosis due to persistent exposure. The literature has reported honeycomb-like interstitial pneumonia and varying degrees of fibrosis in welder autopsy materials (2–4). Both silicosis and TB result in impaired pulmonary function, and both have an appreciable mortality, even when occurring as single disease entities. TB represents a major health hazard to silicotics living in high TB prevalence areas, because of their increased susceptibility to disease. Based on the relationship between silicosis increasing the prevalence of TB among workers, we naturally speculate that the deposition of iron particles in the lungs causes lung function damage and changes in immune response, and iron pneumoconiosis patients are also greatly susceptible to TB. As a result, people engaged in welding and other occupations for a long time need to be well protected to prevent occupational disease and TB, early detection is important for reversible siderosis recovery. As such, differential diagnosis might be difficult. Here, we present a case of iron dust lung in a patient who has no symptoms but has nodular lesions visible on Computed Tomography(CT). The alveolar lavage fluid was examined by various staining methods and pathological examination of lung tissue, to show the different morphological manifestations of the case. All above can improve the morphological identification ability of inspectors and make a quick diagnosis, so as to provide laboratory basis for the prevention and treatment of iron pneumoconiosis.

## Case description

**Figure 1:**
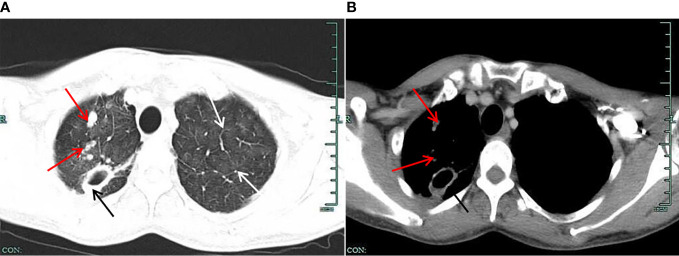
**(A)** Lung window on chest CT. The figure shows the multiple “ground glass” blurred shadows on both lungs (shown by the white arrow); the upper lobe of the right lung shows multiple nodular foci, one of which is larger (shown by the black arrow), showing a thick-walled cavity, and the edge of the lesion has multiple small nodule foci (shown by the red arrow). **(B)** Mediastinal window on chest CT. The figure shows a thick-walled cavity shadow (shown by a black arrow) in a larger nodular foci of the upper lobe of the right lung. The inner wall of the cavity is still smooth, the adjacent pleural traction is changed, the marginal small nodule foci are slightly displayed (shown by the red arrow), and the deposition of granular materials is visible.

**Figure 2:**
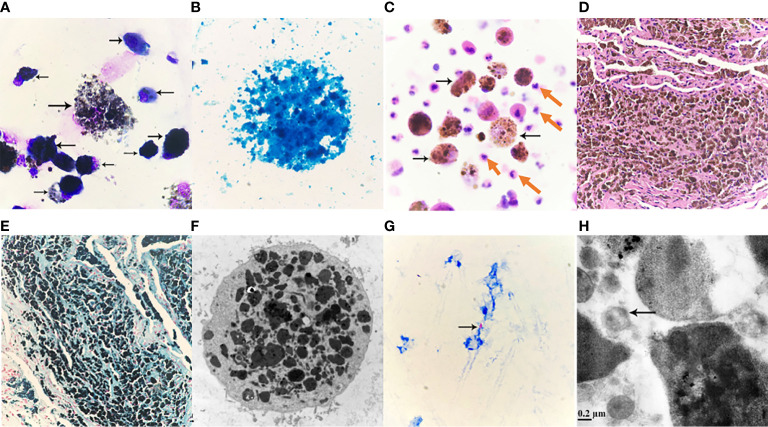
**(A)** Alveolar lavage fluid Wright-Giemsa Stained ×1000. Nucleated cells are significantly increased, easy to see piles, or scattered macrophages, macrophages can be seen round, or circular; soma medium in size, or enlarged; Swallow a large number of black gray, or brown-black particles, different sizes, different shades of coloring, cytoplasmic internal cause is full of a large number of particles, so that the soma swells, cytoplasm is aggregated or scattered black masses, the nucleus is squeezed by particles, distributed at the edge of the cell, or because of particle covering, the structure of the nucleus cannot be seen clearly, macrophage soma that swallows a large number of particles is easy to apoptosis, rupture, nucleus chromatin is loose, granules are scattered due to cell rupture. **(B)** Alveolar lavage solution, Prussian blue staining ×1000. Cytoplasmic macrophages filled with iron granules stained blue. Prussian blue staining positive reaction, a large number of granules covered leading to unclear cell structure. **(C)** Pathological fluid-based Hematoxylin-eosin staining (HE staining) of the posterior right upper lobe of the lung ×400. Significantly increased nucleated cells, scattered cells, evenly distributed, mainly neutrophils (pointed by red arrows), easy to see macrophages (pointed by black arrows), macrophage soma enlarged, round, or circular, it can be seen that macrophages engulf a large number of different sizes, lumpy, or fine, brownish-yellow, brownish-black particles; Macrophages and neutrophils are at different levels of visual field. **(D)** Pathological HE staining of lung tissue slices ×100. Visible lung tissue filled with brownish-yellow or brownish-black particles of varying sizes. **(E)** Lung tissue section, Prussian blue staining ×100. Visible lung tissue cloth granular, lumpy full blue, or blue-black particles, Prussian blue staining positive reaction. **(F)** Alveolar lavage fluid, transmission electron microscopy ×8000. The enlarged macrophage cytoplasm is filled with gray-black iron particles of different sizes and irregular shapes, and the nucleus cannot be seen due to the facet; Marked reduction in organelles may be associated with impaired macrophage immune function. **(G)** Fibrotic brush sample smear, acid-fast stained. Acid-fast bacilli (pointed by the arrow) can be seen: 6 Bars can be seen in 300 fields (×1000). **(H)** Alveolar lavage fluid, transmission electron microscopy ×50,000. *M. tuberculosis* (indicated by arrow).

A 49-year-old male patient who came to our hospital for three weeks of lung shadow found on physical examination had no cough and sputum, no dyspnea, no appetite or weight loss, no fever, chest pain or hemoptysis, no previous diabetes, no history of trauma or radiation exposure. He had surgery for a fracture of the left clavicle five years ago, the pre-operative chest X-ray have not showed any siderosis changes, and postoperative recovery is satisfactory. When he came to our hospital for examination, the patient performed well in all aspects of his body, body temperature: 36.6°C, pulse: 69 times/min, breathing: 20 times/min, blood pressure: 122/82 mmHg. The patient was mind and spirit, with clear breath sounds in both lungs and no obvious rales of dry and wet, and the remaining physical examination revealed no obvious anomalies. There were no significant abnormalities in common laboratory tests. No significant abnormalities were seen in the routine Electrocardiogram (ECG). Examination of epigastric ultrasonography did not suggest abnormalities associated with this disorder. Pulmonary ventilation function measurement: mild obstructive pulmonary ventilation dysfunction, normal lung diffusion function, negative drug relaxation test. The patient has been working as an “electric welder” for more than thirty years and had a history of smoking for well over twenty years, ten cigarettes per day. He quit smoking after lung shadow was revealed in physical examination. Chest CT done three weeks ago suggested diffuse lesions and localized emphysema, thick-walled cavities and multiple nodules seen in both pulmonary and mediastinal windows (**Figures 1A, B**). The diffuse ground-glass changes in two lungs were considered as diffuse alveolar exudation, at the same time under the two pulmonary interstitial changes, combined with occupational history, considering pneumoconiosis may. Although welding-associated pulmonary fibrosis was the most likely diagnosis for this patient, for further differential diagnosis, we communicate with the patient and his family and inform them of the necessity and risks, and after having their consent, we perform a fiberoptic bronchoscopy. We performed fiber bronchoscopy for differential diagnosis and obtained bronchoalveolar lavage fluid from the posterior segment of the right upper lobe and lingual segment of the left upper lobe. Thecell counts and leukocyte classification results: the posterior upper lobe lavage fluid of the right lung had a bloody turbid appearance, a nucleated cell count of 510/μl and neutrophils accounted for 60%, and the appearance of the lingual segment of the left upper lobe is colorless and micro-muted, with a nucleated cell count of 63/μl, neutrophils 6%. The St. Regis staining of Bronohoalveolarlavage (BAL) fluid showed macrophages engulfing a large number of brown-black particles of varying sizes (**Figure 2A**). Positive Prussian blue staining (**Figure 2B**), alveolar lavage fluid pathological fluid base, and lung biopsy (**Figures 2C–E**), is composed of substantial iron particles. Under the electron microscope visible macrophage cytoplasm containing large size, structure, irregular iron particles (**Figure 2F**), which are brown-yellow or brown-black with different sizes, metallic luster, and strong refraction. The patient’s macrophage iron particles differ significantly from those in cases of pulmonary hemorrhage, in which the hemosiderin-containing particles are bluish-black, uneven in size, metallic, and non-refractive. According to pneumoconiosis diagnostic criteria (5), the patient was diagnosed with pneumoconiosis; also chest CT showed wall cavities with mural nodules, multiple nodules around them, and some nodules with calcification, considering the possibility of old TB. Further acid-fast staining of bronchoscopic brush smears and alveolar lavage fluid showed that acid-fast bacilli were positive (6 in 300 fields) (**Figure 2G**). Acid-fast bacilli were also detected by electron microscopy (**Figure 2H**), biopsy of the posterior right upper lobe of the lung is granulomatous inflammation with necrosis, and the patient was diagnosed with siderosis and TB.

The diagnosis of TB in this patient was clear. No obvious abnormalities in Cryptococcus antigen detection, Mycobacterium TB identification, and for rifampicin resistance testing. Mycobacterium tuberculosis and RIF resistance gene testing is detected by the export kit, as long as the sample is added to the kit, and after a period of time, the instrument can display the results. We contacted the patient, learned that he had no family history of TB, and recommended that he go to the local TB hospital for regular chest CT check-ups. The patient also followed our advice to change jobs to avoid further lung damage.

## Literature review and discussions

Pneumoconiosis is a class of diseases caused by the build-up of inhaled dust, which may cause a reaction to the tissues of the lungs. Such diseases exist in two different clinical pathologic forms, one is fibrotic (e.g., silicosis, coal workers’ pneumoconiosis (CWP), asbestosis, beryllium poisoning, and talc), which can be focal nodular or diffuse fibrosis; the other form is nonfibrotic (e.g., siderosis, stannosis, and baritosis), consisting of granules-laden macrophages with very low or no fibrosis (6, 7). The most common type of pneumoconiosis is the fibrotic form of pneumoconiosis, including silicosis, CWP, and asbestosis. Here we discuss the rare siderosis due to welding.

Chronic effects of welding on the lungs include asthma, pneumosiderosis, and relatively rare lung cancer. Iron dust lung, a rare but most common welding-related occupational disease that causes the accumulation of iron particles in alveolar macrophages (8), was originally used by Zenker in 1866 to describe lung pathology caused by long-term sustained inhalation of iron or iron oxide dust (1). It was also described in 1936 by Doig and McLaughlin in a prospective study that examined the clinical and chest X-ray results of 16 welders. In chest X-rays, small circular shadows could be detected, while lung function was not impaired. As in this case, many patients are asymptomatic, and it takes years for them to develop symptoms. The most common complaints are shortness of breath, cough, and sputum production. Doig and McLaughlin followed up 15 of them for nine years, who remained healthy without any respiratory symptoms or dysfunction (4).

Unfortunately, iron has also been proved to cause fibrosis in some cases, especially when mixed with large amounts of sílica. Iron deposition can also lead to changes in symptoms and function of lung. In 1988, Funahashi (9) et al. surveyed 10 symptomatic welders, and histological examination revealed some degree of interstitial pulmonary fibrosis in all cases. Interstitial pulmonary fibrosis is a rare clinical disorder commonly associated with long-term and heavy exposure to welding fumes in poorly ventilated workplaces, which can appear with emphysema, especially in smokers. It is assumed to be a response to iron-containing particles, many of which were found within the fibrotic alveolar septum. The elemental content of these lung tissues was also analyzed by energy-dispersive X-rays, suggesting that the welder’s iron dust lung may be associated with interstitial fibrosis (2, 3, 8).

Epidemiological studies and case reports have confirmed that inhalation of silica dust increases the prevalence of TB among workers, but the magnitude of the impact remains uncertain. Recent findings suggest that patients without silicone lung may be susceptible to TB when exposed to silica, and even if exposure is stopped, the risk appears to be lifelong (6). The mechanism of this relationship is unclear. Is reported in the literature a few possible reasons, such as inhalation of silica altering the immune response of the lungs, impairing the function of macrophages, and causing their apoptosis; an excess of surfactant protein A in BAL fluid appears to be associated with an increased risk of TB; and finally, *M. tuberculosis* can remain dormant within silicone nodules, direct damage of macrophages and the low permeability of drugs in these nodules may lead to subsequent treatment failure and high recurrence rates in these patients (10). It remains unclear whether coal miners with pneumoconiosis are also at risk of TB. A study with 53753 coal miners reported that miners were at three times higher risk of TB than the general population in the same area (11). Since both iron and silicone are pneumoconioses, we reasonably suspect that iron dust lung and TB also have the aforementioned concern.

In ordinary cases, the diagnosis of mycobacterial infection is based on the presence of typical clinical features of TB, such as persistent cough, hemoptysis, weight loss, fever, or changes in imaging findings. Meanwhile, sputum smears and cultures are needed to confirm the disease. However, in patients with pneumoconiosis, due to the deformation of bronchial stenosis and even occlusion caused by the contraction of scars in the lungs, TB bacteria are often not detected in sputum, and even if checked repeatedly, most patients still have negative results. At the same time, in patients with a high staging of pneumoconiosis, imaging often shows complex and variable, the nodules are fused into clumps, showing irregular clumps, or patchy, fibrous cord-like shadows, blurred edges, and have lost the characteristic changes of TB. At this time pneumoconiosis complicated by TB is difficult to distinguish (7, 12).

In this case, a welder with more than 30 years of experience, iron-containing particles were most likely to be inhaled and deposited in the lungs. Similar to previous reports, the patient’s main finding on chest CT was diffuse ground glass-like changes in both lungs, which has been described as the most common finding among welders. These ambiguous micro nodes do not reflect pathologically reactive fibrosis but do correlate with iron-bearing macrophages located in the alveolar space as indicated by Prussian blue staining. In the morphological analysis of alveolar lavage fluid, when macrophages engulf a large number of iron particles, different staining methods and electron microscopy show different morphological characteristics.

**Figure 3:**
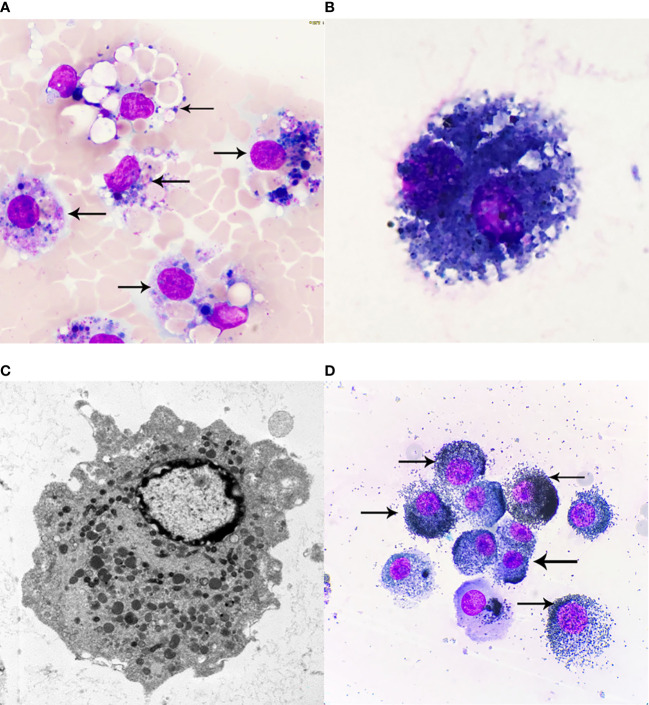
**(A)** Hemosiderin cells, Wright-Giemsa Stained ×1000. Macrophages engulf fresh red blood cells or red blood cell fragments, and after Wright-Giemsa Staining, intracytoplasmic digestion can be seen to form vacuole areas, and blue or blue-black hemosiderin granules of different sizes can also be seen, that is, hemosiderin cells. **(B)** Dust cells, Wright-Giemsa Stained ×1000. Macrophages that mainly engulf substances that cannot be broken down, such as dust. Black particles and droplets of blue lipids formed by tar can be seen. **(C)** Electron microscopy of macrophages ×10000. Macrophages are diverse in morphology and vary due to their different functional states, generally round or oval in shape, with short protrusions, and those with active functions often extend longer pseudopodia and are irregular. The nucleus is small, round or oval in shape, and darkly colored. Under transmission electron microscopy, the cytoplasm contains a large number of primary lysosomes, secondary lysosomes, phagocytosic vesicles and phagosomes, in addition to the more developed Golgi complex, a small number of mitochondria and a rough endoplasmic reticulum. **(D)** Melanoma cells, Wright-Giemsa Stained ×1000. Melanoma cells have moderate to rich cytoplasm and eccentric nuclei, and cell morphology is characteristic of malignant tumor cells, chromatin is loose, and melanoma cells have fine, uniform granules; melanin particles can be found in the cytoplasm of tumor cells or melanoma macrophages. The arrows of **(A)** refer to more typical Hemosiderin cells, the arrows of **(D)** refer to more typical Melanoma cells.

In discussing this case, we should also distinguish between siderosis, desquamative interstitial pneumonia, and idiopathic pulmonary hemosiderosis. In most cases, desquamative interstitial pneumonia is associated with smoking, and its histological manifestations include involvement of parenchymal lobes and significant accumulation of alveolar macrophages, but macrophage Prussian blue stain is negative. While hemoptysis, iron deficiency anemia and a triple sign of lung parenchymal infiltration on chest x-ray are three of the hallmarks of idiopathic pulmonary hemosiderosis, which often occurs in children. Our diagnosis can exclude desquamative interstitial pneumonia and idiopathic pulmonary hemosiderosis because macrophage iron stain is positive and there is no iron deficiency anemia (2). In addition, morphologically, we also need to identify between iron-containing granule cells, dust cells, hemosiderin cells, and melanocytes (**Table 1**). Iron particles in iron-containing granule cells have a brownish-yellow metallic luster, strong refractive index, and vary in size (13, 14); hemosiderin cells and dust cells belong to macrophages, but the contents of the phagocytosis are different. Hemosiderin-containing cells are the macrophage that engulfs erythrocytes. Hemoglobin broken down to form hemosiderin granules suggestsold bleeding, more common in bloody specimens. Hemosiderin that truly comes from alveolar hemorrhage is usually blue-black and lumpy, with a single block reaching a diameter of 7 to 8 μm (15) (**Figure 3A**), fresh red blood cells or old red blood cells can be seen in the background, and phagocytes or red blood cell debris in the macrophage cytoplasm can also be seen. Dust cells are formed by engulfing non-decomposable substances such as dust (**Figure 3B**), the particles are mostly dark black in size and more common seen in patients who smoke, and also in patients with occupational pneumoconiosis. Besides black particles, blue lipid droplets formed by tar can also be seen in the dust cells of patients who smoke. Under electron microscopy, the particles of both types of cells are black (**Figure 3C**) and need to be identified by other methods. Melanoma cells (**Figure 3D**) have moderate to rich cytoplasm and eccentric nuclei, cell morphology is characteristic of malignant tumor cells, chromatin is loose, and cells have fine and uniform granules. Melanin particles can be found in the cytoplasm of tumor cells or melanoma macrophages and may be indistinguishable from hemosiderin. The latter is usually coarser and more refractive than melanin particles, and histochemical staining (Fontana-Masson for the diagnosis of melanoma and Perls for hemosiderin) may be helpful. The immunochemistry of melanoma cell markers also contributes to the diagnosis of melanoma (16).

**Table 1:** Cytological characteristics of iron-containing granules, dust cells, hemosideranocytes, and melanocytes, differences in particles in cells, and suggestive diseases.

Cells	Cytologic features	Characteristics of particles in cells	Diseases
Iron-containing granule cells	The main component are macrophages that digest iron fragments (macrophages engulf foreign iron particles that cannot be digested and absorbed)	Blue-black and black iron particles of different sizes, with metallic luster and a high index of refraction, there are many large pieces of iron in the macrophages	Siderosis
Dust cells	Macrophages that mainly engulf substances that cannot be broken down, such as dust	Black particles and droplets of blue lipids formed by tar	Pneumoconiosis/smoking
Hemosiderin cells	Metabolites after macrophages engulf red blood cells or red blood cell fragments are hemosiderosin granules	Blue-black and uniform in size without metallic luster and refraction	Pulmonary hemorrhage
Melanoma cells	Mesothelial-like cells with moderate to abundant cytoplasm and eccentric nuclei with characteristics of tumor cells	Black, finer and more uniform than iron particles, less refractive	Melanoma

## Conclusions

In summary, this report describes a case of iron dust lung with concomitant TB. To avoid further lung injury, such patients should be discontinued from occupational exposure. Regular and long-term monitoring is required for patients with a history of exposure to welding fumes, as the risk of TB in this group is significantly elevated, and this risk may be long-standing even if exposure is stopped. As with our patient, any typical but non-specific features of TB in patients with siderosis should be thoroughly examined, such as persistent cough, weight loss, and fever, to rule out TB. In addition, any radiological findings, such as asymmetrical nodules, rapid progression, or cavities, should be of concern. TB treatment in these patients may be prolonged because sputum specimens may remain smear-positive for a longer period. Finally, patients with siderosis should receive close and regular follow-up for early detection of any new TB cases or recurrences. When clinicians encounter such rare cases, while referring to the patient’s symptoms and imaging performance, a detailed understanding of the work history, combined with a variety of morphological results, such as conventional morphological examination of alveolar lavage fluid, alveolar lavage fluid pathological fluid-based examination, lung tissue biopsy and Prussian blue staining, etc., will be very helpful for diagnosis and differential diagnosis.

## Data availability statement

The datasets presented in this study can be found in online repositories. The names of the repository/repositories and accession number(s) can be found in the article/supplementary material.

## Ethics statement

The studies involving human participants were reviewed and approved by the Ethics Committee of Hangzhou First People’s Hospital, Zhejiang University School of Medicine. The patients/participants provided their written informed consent to participate in this study. Written informed consent was obtained from the individual(s) for the publication of any potentially identifiable images or data included in this article.

## Author contributions

YZ: Case collation and writing of thesis. YW: Transmission electron microscopy experiment and electron microscopy morphology guidance. WL: Writing of thesis and editing of language. HW: Imaging and diagnosis. DH: Experiment planning and arrangement. JJ: Thesis writing planning and guidance. QL: Histopathological biopsy and diagnosis. JL: Experimental preparation and operation of transmission electron microscopy. QC: Culture, identification and morphological analysis of pathogenic microorganisms. SR: Experimental operation of morphological inspection of irrigation liquid. JT: Experimental arrangement and guidance for thesis writing. SW: Experimental data sorting, analysis and literature retrieval. All authors contributed to the article and approved the submitted version.
