# Erratum: Case Report: application of morphology in the diagnosis of siderosis in a patient with tuberculosis infection

**DOI:** 10.3389/fonc.2023.1281636

**Published:** 2023-09-26

**Authors:** Frontiers Production Office

**Affiliations:** Frontiers Media SA, Lausanne, Switzerland

**Keywords:** welder, siderosis, tuberculosis, morphology, case report

Due to a production error, there was a mistake in **Table 1** as published. The second and third rows of the table were duplicated. The corrected **Table 1** appears below. The publisher apologizes for this mistake.

**Table 1:** Cytological characteristics of iron-containing granules, dust cells, hemosideranocytes, and melanocytes, differences in particles in cells, and suggestive diseases.

Cells	Cytologic features	Characteristics of particles in cells	Diseases
Iron-containing granule cells	The main component are macrophages that digest iron fragments (macrophages engulf foreign iron particles that cannot be digested and absorbed)	Blue-black and black iron particles of different sizes, with metallic luster and a high index of refraction, there are many large pieces of iron in the macrophages	Siderosis
Dust cells	Macrophages that mainly engulf substances that cannot be broken down, such as dust	Black particles and droplets of blue lipids formed by tar	Pneumoconiosis/smoking
Hemosiderin cells	Metabolites after macrophages engulf red blood cells or red blood cell fragments are hemosiderosin granules	Blue-black and uniform in size without metallic luster and refraction	Pulmonary hemorrhage
Melanoma cells	Mesothelial-like cells with moderate to abundant cytoplasm and eccentric nuclei with characteristics of tumor cells	Black, finer and more uniform than iron particles, less refractive	Melanoma

The original version of this article has been updated.

